# Modified Continuum Mechanics Modeling on Size-Dependent Properties of Piezoelectric Nanomaterials: A Review

**DOI:** 10.3390/nano7020027

**Published:** 2017-01-26

**Authors:** Zhi Yan, Liying Jiang

**Affiliations:** 1 Department of Mechanics, Huazhong University of Science and Technology, Wuhan 430074, China; 2 Hubei Key Laboratory of Engineering Structural Analysis and Safety Assessment, Luoyu Road 1037, Wuhan 430074, China; 3 Department of Mechanical and Materials Engineering, The University of Western Ontario, London, ON N6A 5B9, Canada; lyjiang@eng.uwo.ca

**Keywords:** piezoelectric nanomaterials, continuum mechanics modeling, size-dependent properties, surface effects, flexoelectricity, non-local theory

## Abstract

Piezoelectric nanomaterials (PNs) are attractive for applications including sensing, actuating, energy harvesting, among others in nano-electro-mechanical-systems (NEMS) because of their excellent electromechanical coupling, mechanical and physical properties. However, the properties of PNs do not coincide with their bulk counterparts and depend on the particular size. A large amount of efforts have been devoted to studying the size-dependent properties of PNs by using experimental characterization, atomistic simulation and continuum mechanics modeling with the consideration of the scale features of the nanomaterials. This paper reviews the recent progresses and achievements in the research on the continuum mechanics modeling of the size-dependent mechanical and physical properties of PNs. We start from the fundamentals of the modified continuum mechanics models for PNs, including the theories of surface piezoelectricity, flexoelectricity and non-local piezoelectricity, with the introduction of the modified piezoelectric beam and plate models particularly for nanostructured piezoelectric materials with certain configurations. Then, we give a review on the investigation of the size-dependent properties of PNs by using the modified continuum mechanics models, such as the electromechanical coupling, bending, vibration, buckling, wave propagation and dynamic characteristics. Finally, analytical modeling and analysis of nanoscale actuators and energy harvesters based on piezoelectric nanostructures are presented.

## 1. Introduction

Piezoelectricity represents the capability of some materials to convert mechanical energy into electrical energy and vice versa. Piezoelectricity was first discovered by brothers Pierre and Jacques Curie in 1880 [1]. There are many materials that naturally possess piezoelectricity, such as tourmaline, Rochelle salt, topaz, quartz, cane sugar, etc. The first important application of piezoelectric materials was to generate acoustic waves by crystal quartz, which lead to the development of various piezoelectric transducers for the purpose of military applications during the first half of the 20th century [2]. Besides the natural piezoelectric crystals mentioned above, an important class of piezoelectric materials commonly used nowadays are called piezoelectric ceramics, which were synthesized in the 1950s. These synthetic materials such as lead zirconate titanate (PZT), barium titanate (BaTiO${}_{3}$) and lead titanate (PbTiO${}_{3}$) can be tailored for particular applications according to their specific properties. In the 1980s, the idea of micro-electro-mechanical-systems (MEMS) emerged to meet the requirements of miniaturization and integration of electronic components. Consequently, piezoelectric MEMS devices such as switches, resonators, filters and transformers were developed, which served in robotics as well as everyday applications [3,4]. With the miniaturization of various functional devices, piezoelectric nanomaterials (PNs) have attracted increasing interest during the last decade. It is worth mentioning the pioneer work of Professor Zhonglin Wang [5], who demonstrated the first piezoelectric nanogenerator prototype using a single zinc oxide (ZnO) nanowire in 2006. Due to the remarkable progress in the research and the advances of synthesis techniques, researchers have been able to synthesize PNs with various materials and configurations. Examples of PNs with a perovskite crystal structure include zero-dimensional bismuth ferrite (BiFeO${}_{3}$) nanoparticles [6], one and two dimensional PZT nanofibers/films/ribbons [7,8,9] and BaTiO${}_{3}$ nanowires/films [10,11]. With combined piezoelectricity and ferroelectricity, these perovskite PNs are promising for constructing ferroelectric thin film memory devices [12,13] and piezoelectric nanogenerators [9,11]. Other kinds of PN with a wurtzite crystal structure, such as ZnO, gallium nitride (GaN), zinc sulfide (ZnS), have also received considerable attention in recent years. Due to the electrostatic interaction energy and distinct chemical activities of the polar surfaces, a wide range of configurations can be easily formed for this group of materials, including ZnO and GaN nanoparticles/wires/tubes/belts [14,15,16,17,18,19,20]. The unique piezoelectric and semiconducting properties of these novel materials have made them ideal candidates as building blocks and functional elements in nanopiezotronics, such as lasers [21], resonators [22], field-effect transistors [23], diodes [24], strain sensors [25], strain-controlled logic gates [26], energy generators [27] and photovoltaic devices [28]. Wang [29] has given a comprehensive review on the applications of one-dimensional ZnO nanomaterials in electromechanical coupled devices. It is worth mentioning that the piezoelectricity has also been discovered in the boron nitride (BN) nanosheets/tubes and monolayers of materials belonging to the family of TMDC (transition metal dichalcogenide), as reviewed in reference [30]. Furthermore, piezoelectric polymers such as polyvinylidene difluoride (PVDF) are attractive for building energy harvesters due to their high flexibility and stretchability, biocompatibility and low cost [31]. To achieve the full potential functionality of these novel nanomaterials, it is necessary to have a better understanding of the underlying fundamentals of their unique physical and mechanical properties.

The mechanical and physical properties of bulk piezoelectric materials have been well studied in the literature. However, “small is different”, and the corresponding properties of PNs can differ dramatically from those of their bulk counterparts. Indeed, many applications of PNs mentioned above become possible since the material properties at the nanoscale are improved. Therefore, it is important to characterize the size-dependent properties of PNs. From the experimental perspective, micro-electro-mechanical-systems (MEMS) in situ transmission electron microscopy (TEM) [32,33] or in situ scanning electron microscopy (SEM) [34] has been successfully adopted to characterize the mechanical properties of PNs while the piezoelectric properties of some materials have been studied by using the piezoelectric force microscopy (PFM) [35,36]. There is a general agreement concluded from the experiments that the properties of PNs are size-dependent, e.g., as the nanowire diameter decreases from 80nm to 20nm, the Young’s modulus of ZnO nanowire increases from ∼140 to 160 GPa [32]; the fracture strain and strength of ZnO nanowires also increase as the nanowire diameter decreases [34]; the piezoelectric constants of GaN nanowires were reported up to six times that of the bulk values [37]. It was also noted that the piezoelectric coefficient of ZnO nanobelt is frequency dependent [35,38], which is the so-called retardation or relaxation behavior and could be explained by the Debye model [38]. Meanwhile, atomistic simulations have been extensively used to predict the elastic and piezoelectric properties of PNs. However, it should be highlighted that simulation results may show size-dependence with much smaller nanowires. As an example, Chen et al. [39] predicted that the elastic properties of ZnS nanowire with sizes of ∼5 nm would probably agree with that of bulk ZnS by using the first-principles approaches. Zhang et al. [40] studied the influence of tensile and compressive strain on the ferroelectric behaviors of BaTiO${}_{3}$ nanowires based on a molecular dynamics approach. The size-dependence of the piezoelectric coefficient of the BaTiO${}_{3}$ nanowires was evidenced from this study, and the piezoelectric coefficient of the nanowire approaches the bulk value ($e_{33}=6.71\;C/m^{2}$) of the BaTiO${}_{3}$ when its diameter is larger than 2.4nm. First principles-based density function theory calculations also showed that, for both ZnO and GaN nanowires, a significant enhancement, i.e., approximately two orders of magnitude, in piezoelectric coefficient could be achieved if the diameter of the nanowire were reduced to below 1nm [41]. Utilizing the molecular dynamics technique, Momeni et al. [42] found that the piezoelectric coefficients of ZnO nanobelts with lengths of 150.97 Å increase when the lateral sizes reduce from 37.37 to 8.13 Å. It was also interesting to find that the elastic and piezoelectric modulus of ZnO nanowires are enhanced with diameter d>1nm, but are dropped suddenly for d<1nm due to the phase transition from nanowires to nanotubes, and the degradation of the mechanical properties of ZnO nanobelts with lateral dimension <1nm was also observed [43]. A comprehensive review of the mechanical and piezoelectric properties of wurtzite piezoelectric nanowires has been carried out by Espinosa et al. [44]. These existing studies have clearly evidenced the size-dependent material properties of piezoelectric nanostructures. Nevertheless, there are some limitations for both the experimental characterization and the atomistic simulations. In general, the extremely small sizes of nanostructures can impose serious challenges for experimental measurements. For example, it is noted that uncertainties in boundary conditions, metrology of the cross-section, instrument calibration and sample manipulation often lead to significant scatter in experimental data [32]. In addition, the mechanical and electromechanical properties of PNs may be significantly affected by certain defects, which makes the interpretation of measured data more difficult. For the atomistic simulations, investigations are limited by computation capabilities at both the length and the time scales, considering the large number of atoms in a typical nanostructure. Moreover, the semi-empirical force fields employed in the molecular simulations should be validated against the first-principles calculations. As an alternative way, many researchers have resorted to the continuum mechanics modeling to investigate the properties of PNs due to its superior computational efficiency and robustness. However, the conventional continuum models ignore the variation of interatomic quantities, and thus fail to capture the size effects of nanostructures. Therefore, new continuum modeling techniques incorporating the nanoscale structure features must be developed to overcome this obstacle.

It is commonly believed that surface effects are attributed to the size-dependent properties of nanomaterials due to their large surface-to-volume ratio. As the structural size reduces to the nanoscale, the number of atoms at the surface increases in respect to that in the bulk. Since the surface atoms are more unstable than the bulk ones, they may induce the unique properties of nanomaterials. In addition, strain gradient (or nonuniform deformation) induced flexoelectricity, which is a more universal and diverse electromechanical coupling effect in comparison with piezoelectricity, is expected to contribute to the size-dependent properties of PNs and explain the unusual ferroelectric properties of materials such as the unusual domain structure and domain wall observed at the nanoscale. It should be mentioned that the domain patterns are important in determining the properties such as the piezoelectric coefficient of ferroelectrics [45], and it has been theoretically justified that the engineered domain configurations can be made use of to improve the performance of actuators, sensors and energy harvesting devices [46,47]. With the consideration of surface effects or flexoelectricity, a number of modified continuum models have been established to explore the mechanical, physical, and electromechanical coupling properties of PNs. Extended from the non-local elasticity [48], a non-local piezoelectricity theory has also been developed to analyze the mechanical behaviors of PNs. A review of these theories as well as the size-dependent electromechanical coupling, bending, vibration, buckling, wave propagation and dynamic characteristics of PNs are reviewed in the following sections. The applications of nanostructured piezoelectric materials as actuators and energy harvesters will also be discussed.

## 2. Novel Modified Continuum Theories for PNs

### 2.1. Surface Piezoelectricity Theory

**Figure 1:**
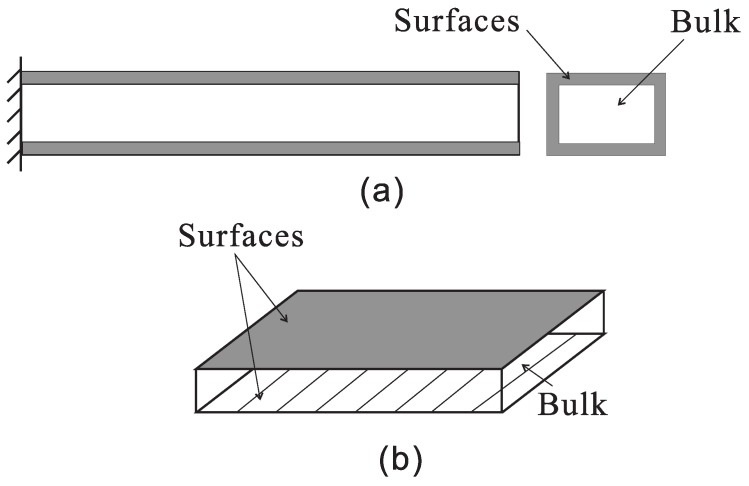
Schematic of (**a**) a piezoelectric nanobeam; and (**b**) a piezoelectric nanoplate with surface effects.

Since the atoms at and near a free surface or an interface experience a different local environment compared with those in the bulk of a material due to the reduced coordination, the equilibrium position and the energy associated with these atoms are generally different from those of the atoms in the bulk [49]. Therefore, the creation of a surface leads to excess free energy in a solid, i.e., the surface free energy, which is the origin of the surface effects. As the size of materials reduces to the nanoscale, the influence of the surface or interface is substantially enhanced due to the increased surface-to-volume ratio. Thus, the surface effects should be incorporated into the continuum modeling of PNs to account for the size-dependent properties. Gurtin and Murdoch [50] proposed a surface elasticity model with the theoretical framework stemming from the continuum mechanics. In this model, the surface is regarded as a thin layer with negligible thickness adhered to the underlying bulk material without slipping. The surface properties and constitutive relations are different from those of the bulk, and the equilibrium of the surface is governed by the generalized Young–Laplace equations. As an extension of the surface elasticity model, Huang and Yu [51] proposed a surface piezoelectricity model by assuming that the surface energy density depends on the electric field at the surface and the in-plane strains. Based on this assumption, the surface stresses $\sigma_{\alpha \beta }^{s}$ and the surface electric displacements $D_{i}^{s}$ can be expressed as

(1a) $$\sigma_{\alpha \beta }^{s}=\sigma_{\alpha \beta }^{0}+c_{\alpha \beta \gamma \delta }^{s}\varepsilon_{\gamma \delta }-e_{\alpha \beta \kappa }^{s}E_{k}$$

(1b) $$D_{i}^{s}=D_{i}^{0}+e_{\alpha \beta i}^{s}\varepsilon_{\alpha \beta }+\kappa_{ij}^{s}E_{j}$$

with $c_{\alpha \beta \gamma \delta }^{s}$, $e_{\alpha \beta k}^{s}$ and $\kappa_{ij}^{s}$ being the surface elastic coefficients, the surface piezoelectric coefficients and the surface dielectric coefficients, respectively. $\sigma_{\alpha \beta }^{0}$ and $D_{i}^{0}$ are the residual surface stress constants and the residual surface electric displacement constants without any applied strain and electric field. Since most piezoelectric nanodevices are beam- or plate-like structure based, Yan and Jiang [52,53] developed modified piezoelectric nanobeam and nanoplate models by combining conventional Euler beam and Kirchhoff plate theories with the surface piezoelectricity theory. Zhang et al. [54] developed a two-dimensional theory for piezoelectric nanoplates considering surface effects, and their model can describe the extension, flexure and thickness-shear modes of deformation. Zhang et al. [55] also presented general equations of two-dimensional piezoelectric shells with nano-thickness in an orthogonal curvilinear coordinate system with the surface effects. In 2010, Shen and Hu [56] established a theoretical framework of nanoscale dielectrics, in which the total internal energy density of the material (*U*) was calculated as the sum of the surface energy density ($U_{s}$) and the bulk energy density ($U_{b}$). It should be noted that such a "bulk + surface" model has been widely employed to represent the piezoelectric nanostructures in the modelling, as illustrated in Figure 1. For a one-dimensional nanobeam, a circumferential surface is considered, as seen from Figure 1a. While, for a two-dimensional nanoplate, only the upper and lower surfaces are considered due to the small plate thickness, as demonstrated by Figure 1b. By considering the equilibrium of a small element of a curved interface in elastic solids, the generalized Young–Laplace equations were derived [57], which indicate that traction jumps will be induced by the existence of the surface stresses, i.e.,

(2) $$\left[\sigma^{+}-\sigma^{-}\right]\cdot n=-\nabla_{S}\cdot \sigma^{s}$$

Similarly, an electric displacement jump exists for piezoelectric nanomaterials due to the surface piezoelectricity, i.e.,

(3) $$\left[D^{+}-D^{-}\right]\cdot n=-\nabla_{S}\cdot D^{s}$$

These two equations constitute the surface effects for the PNs. It should also be mentioned that the bulk material possesses the same constitutive relations as the traditional piezoelectric materials.

### 2.2. Theory of Flexoelectricity

Flexoelectricity is a universal electromechanical coupling in all dielectrics including soft matter, polymers and hard materials. It refers to a spontaneous electric polarization in response to nonuniform strains (or strain gradients). In a phenomenological way, the electric polarization $P_{i}$ can be expressed in terms of strain gradients $\varepsilon_{jk,l}$ as

(4) $$P_{i}=\mu_{ijkl}\varepsilon_{jk,l}$$

Obviously, the strength of this flexoelectric effect depends on both the flexoelectric coefficients $\mu_{ijkl}$ and the strain gradients. In general, the flexoelectric coefficients are small values, which represent relatively weak electromechanical coupling as compared to the traditional piezoelectricity, particularly for the macroscale materials. This is the main reason that the flexoelectricity has received very limited attention for a long period of time after its discovery in the 1950s. However, the strain gradients are inversely proportional to the scale length of a structure under the same mechanical loads. Therefore, the flexoelectricity is a size-dependent property and becomes more prominent at the nanoscale, which could significantly influence the electromechanical coupling behavior of PNs. Due to the advances in nanotechnology and the potential applications of nanomaterials in electromechanical transduction technology, there has been a trend of increasing scientific interest in the flexoelectricity in recent years. Meanwhile, the flexoelectric constants of certain ferroelectric materials with high dielectric permittivities were measured and found to be remarkably large. Thus, flexoelectricity has gained wide attention from the research community to investigate the electromechanical couplings of PNs. For the fundamentals and recent advances of flexoelectricity, refer to the following reviews [58,59,60,61,62]. In the current paper, we will focus on the efforts in establishing the theoretical framework for dielectrics in order to quantitatively understand the underlying physics of their electromechanical coupling at the nanoscale. For example, Catalan et al. [63] presented a phenomenological model for ferroelectric thin films and accounted for the flexoelectricity by modifying the conventional Landau–Ginzburg–Devonshire (LGD) free energy expression. According to the modified LGD theory, a phase-field modeling approach was also developed to examine the role of the flexoelectric effect in the complex domain patterns in ferroelectric ceramics [64]. Based on the variational principle, Maranganti et al. [65] developed a general framework for dielectrics including the flexoelectric effect. For an isotropic centrosymmetric continuum medium, they also provided Green’s function solutions for the governing equations. Shen and Hu [56] later established a more comprehensive framework for the nanoscale dielectrics by incorporating the flexoelectric effect into the constitutive equations as

(5a) $$\sigma_{ij}=c_{ijkl}\varepsilon_{kl}+e_{ijkl}P_{k,l}+d_{ijk}P_{k}+r_{ijklm}\varepsilon_{kl,m}$$

(5b) $$\tau_{ijm}=f_{ijmk}P_{k}+r_{klijm}\varepsilon_{kl}+\eta_{klijm}P_{k,l}+g_{ijmknl}\varepsilon_{kn,l}$$

(5c) $$E_{i}=a_{ij}P_{j}+d_{jki}\varepsilon_{jk}+h_{ijk}P_{j,k}+f_{jkli}\varepsilon_{jk,l}$$

(5d) $$V_{ij}=b_{ijkl}P_{k,l}+e_{klij}\varepsilon_{kl}+h_{kij}P_{k}+\eta_{ijkmn}\varepsilon_{km,n}$$

where $\sigma_{ij}$, $E_{i}$, $P_{i}$ and $\varepsilon_{ij}$ are traditional stresses, electric fields, polarizations and strains, respectively, with the subscript comma indicating differentiation with respect to the spatial variables. $c_{ijkl}$, $d_{ijk}$ and $a_{ij}$ are the elastic coefficients, piezoelectric coefficients and dielectric susceptibilities as in the traditional linear piezoelectricity. $f_{ijkl}$ and $e_{ijkl}$ are the direct and converse flexcoupling coefficients. $h_{ijk}$, $b_{ijkl}$, $r_{ijklm}$, $\eta_{ijkmn}$ and $g_{ijmknl}$ are the tensors representing the other higher-order couplings, i.e., polarization–polarization gradient, polarization gradient–polarization gradient, strain–strain gradient, polarization gradient–strain gradient and strain gradient–strain gradient. In fact, $e_{ijkl}$ was introduced by Mindlin [66] in his polarization gradient theory while $g_{ijklmn}$ was introduced in the strain–gradient elasticity theory and could represent the non-local elastic effect. $\tau_{ijm}$ and $V_{ij}$ are the higher-order stresses and higher-order local electric fields, which are caused by the flexoelectric effect and some other higher-order coupling effects.

An alternative form of the constitutive equations for dielectrics with the incorporation of the flexoelectricity is given by [67]

(6a) $$\sigma_{ij}={\bar{c}}_{ijkl}\varepsilon_{kl}-{\bar{e}}_{ijkl}E_{k,l}-{\bar{d}}_{ijk}E_{k}+r_{ijklm}\varepsilon_{kl,m}$$

(6b) $$\tau_{ijm}=-{\bar{f}}_{ijmk}E_{k}+r_{klijm}\varepsilon_{kl}-{\bar{\eta }}_{klijm}E_{k,l}+g_{ijmknl}\varepsilon_{kn,l}$$

(6c) $$D_{i}={\bar{a}}_{ij}E_{j}+{\bar{d}}_{jki}\varepsilon_{jk}+{\bar{h}}_{ijk}P_{j,k}+{\bar{f}}_{jkli}\varepsilon_{jk,l}$$

(6d) $${\bar{V}}_{ij}={\bar{b}}_{ijkl}E_{k,l}+{\bar{e}}_{klij}\varepsilon_{kl}+{\bar{h}}_{kij}E_{k}+{\bar{\eta }}_{ijkmn}\varepsilon_{km,n}$$

where pairs of conjugate variables ($D_{i}$, $E_{i}$) instead of ($E_{i}$, $P_{i}$), and ($E_{i,j}$, ${\bar{V}}_{ij}$) instead of ($P_{i,j}$, $V_{ij}$) are employed, and $D_{i}$ is the electric displacement vector. In addition, ${\bar{c}}_{ijkl}$, ${\bar{d}}_{ijk}$ and ${\bar{a}}_{ij}$ are the elastic, piezoelectric and dielectric coefficients different from $c_{ijkl}$, $d_{ijk}$ and $a_{ij}$. ${\bar{f}}_{ijkl}$ and ${\bar{e}}_{ijkl}$ are the direct and the converse flexoelectric coefficients, representing the strain gradient and the electric field coupling, and the electric field gradient and the elastic strain coupling, respectively. ${\bar{h}}_{ijk}$, ${\bar{b}}_{ijkl}$ and ${\bar{\eta }}_{ijkmn}$ represent the electric field–electric field gradient, electric field gradient–electric field gradient and electric field gradient–strain gradient coupling tensors, respectively. From these constitutive relations, it is clearly seen that the flexoelectric effect is characterized by a fourth-order flexocoupling or flexoelectric coefficient tensor. A few efforts have been devoted to determining the nonzero components of these tensors, both from experimental characterization [68] and atomistic simulations [69]. In 2011, the number of independent components of the flexoelectric coefficient tensor for a certain symmetry class of materials was demonstrated by Le Quang and He [70]. Shu et al. [71] determined the flexoelectric coefficient components in a matrix form for materials belonging to specific point groups and Curie groups. Subsequently, Yan and Jiang [72,73] and Zhang et al. [74] developed modified piezoelectric nanobeam and nanoplate models with the flexoelectric effect to investigate the static and dynamic behaviors of PNs. It is also worth mentioning that the flexoelectricity will result in modified boundary conditions of the piezoelectric nanostructures, and the modified elastic boundary conditions were demonstrated by the work of Yurkov [75]. Recently, Yan and Jiang [76] further presented the modified elastic boundary conditions in the cylindrical coordinate system in the presence of the direct flexoelectricity. It should be noted that the flexoelectricity is also important for a special class of soft materials: biological membranes. Deng et al. [77] demonstrated that the electrets and the flexoelectricity permitted the engineering of a rather large electromechanical coupling in soft materials.

### 2.3. Non-Local Piezoelectricity Theory

In order to capture the size-dependent properties of nanomaterials, higher-order continuum mechanics theories such as polarization gradient theory [66], strain gradient theory [78], non-local theory [48], micropolar theory [79] and couple stress theory [80,81,82] have been employed in the literature. Extended from the non-local elasticity, the non-local piezoelectricity theory states that the stresses and the electric displacements at a reference point x depend not only on the strains and the electric fields at the same point but also on all other points $x^{\prime }$ of the body. The non-local constitutive equations can be expressed as

(7a) $$\sigma_{ij}^{nl}\left(x\right)=\int_{V}\alpha \left(|x^{\prime }-x|\right)\sigma_{ij}^{l}\left(x^{\prime }\right)dV\left(x^{\prime }\right)$$

(7b) $$D_{k}^{nl}\left(x\right)=\int_{V}\alpha \left(|x^{\prime }-x|\right)D_{k}^{l}\left(x^{\prime }\right)dV\left(x^{\prime }\right)$$

where $\alpha (|x^{\prime }-x|)$ is the non-local modulus satisfying $\int_{V}\alpha (|x^{\prime }-x\left|\right)dV\left(x^{\prime }\right)=1$, and the volume integral is over the region V occupied by the body. $\sigma_{ij}^{nl}$ and $\sigma_{ij}^{l}$ are, respectively, the components of the non-local stress tensor and the classical stress tensor; $D_{k}^{nl}$ and $D_{k}^{l}$ are the components of the non-local and the classical electric displacement. The classical stress and electric displacement satisfy the traditional constitutive equations as $\sigma_{ij}^{l}\left(x^{\prime }\right)=c_{ijkl}\varepsilon_{kl}\left(x^{\prime }\right)-e_{kij}E_{k}\left(x^{\prime }\right)$ and $D_{k}^{l}\left(x^{\prime }\right)=e_{kij}\varepsilon_{ij}\left(x^{\prime }\right)+\kappa_{ki}E_{i}\left(x^{\prime }\right)$ with $c_{ijkl},e_{kij}$ and $\kappa_{ki}$ being the elastic, piezoelectric and dielectric constants. According to Eringen [48], the non-local effect represented by Equation (7a) can be rewritten in the differential format, i.e.,

(8) $$\left(1-\mu \nabla^{2}\right)\sigma_{ij}^{nl}=\sigma_{ij}^{l}$$

where $\mu ={\left(e_{0}a\right)}^{2}$ is the non-local parameter with *a* being an internal characteristic length (e.g., lattice parameter, granular size) and $e_{0}$ being a material property determined by experimental results or comparison with calculations based on lattice dynamics; $\nabla^{2}$ is the Laplacian operator. Similarly, Equation (7b) can be rewritten as

(9) $$\left(1-\mu \nabla^{2}\right)D_{k}^{nl}=D_{k}^{l}$$

The non-local piezoelectricity theory was firstly employed to solve the crack problems in piezoelectric materials [83], and has been extensively adopted to study the size-dependent mechanical and physical behaviors of PNs in recent years.

## 3. Size-Dependent Properties of PNs

In the literature, the above-mentioned modified continuum theories have been employed extensively to study the size-dependent electromechanical coupling and mechanical properties of PNs. In this section, the electromechanical coupling, bending, vibration, buckling, wave propagation and dynamic characteristics of various piezoelectric nanostructures are reviewed, and a summary of the relative works is demonstrated in Table 1.

### 3.1. Electromechanical Coupling Behaviors of PNs

The electromechanical coupling (EMC) coefficient measures the effectiveness of the EMC and thus is commonly adopted as an important parameter for characterizing the performance of piezoelectric energy harvesting. The EMC coefficient can be obtained through measuring the variation of the energy stored in the electromechanical structure with the change of the electric boundary conditions. Considering the surface effects, this coefficient for a piezoelectric nanobeam $\xi_{eff}$ was determined based on the surface piezoelectricity theory as $\xi_{eff}=\frac{e_{31}^{2}bh+e_{31}^{s}e_{31}\left(2h+6b\right)}{\left(c_{11}\kappa_{33}+e_{31}^{2}\right)bh+\left(c_{11}^{s}\kappa_{33}+e_{31}^{s}e_{31}\right)\left(2h+6b\right)}$ [52] with $c_{11},e_{31}$ and $\kappa_{33}$ being the conventional elastic, piezoelectric and dielectric constants, with $c_{11}^{s}$ and $e_{31}^{s}$ being the surface elastic and piezoelectric constants, and *b* and *h* representing the width and thickness of the beam. It is explicitly demonstrated by this expression that the EMC coefficient varies with the size and can be dramatically enhanced with the decrease of the beam thickness due to the surface effects. Later, the non-local piezoelectricity theory in addition to the surface effects were considered to study the EMC coefficient by Wang and Wang [84], and they found that the EMC coefficient increases, with the increase of the nanobeam thickness, to a peak, and then decreases, which indicates that the non-local parameter *μ* decreases the EMC coefficient. The explicit expression of the EMC coefficient has also been obtained by considering both the surface effects and the flexoelectricity [85], which indicates that the EMC coefficient is enhanced by the flexoelectricity. In addition, the flexoelectricity was also found to enhance the effective piezoelectric constants of a piezoelectric nanowire [86].

The electromechanical coupling fields of both flat structures [52,53] and curved structures including a piezoelectric nanoring [51] and a curved piezoelectric nanobeam [87] have been investigated by using the surface piezoelectricity model. These works indicated that surface effects have a great influence upon the electroelastic fields of the piezoelectric nanostructures. An analytical solution was also obtained for the piezoelectric potential generated in a cantilevered ZnO nanowire with the consideration of the flexoelectricity, indicating the size-dependent electromechanical coupling due to the inhomogeneous strains [88]. Furthermore, the influence of the direct flexoelectricity and the non-local elastic effect on the electroelastic fields of a hollow piezoelectric nanocylinder was investigated by Yan and Jiang [76], in which the flexoelectricity was found to induce clear electroelastic responses that could not be achieved through pure piezoelectricity.

### 3.2. Bending, Vibration and Buckling Behaviors of PNs

The mechanical properties of PNs including bending, vibration and buckling behaviors are crucial for the rigidity and functionality of structures in use, and thus the focus of many investigations. Based on the surface elasticity model, Wang and Feng [89] investigated the surface stress effect on the vibration and buckling of piezoelectric nanowires, and they showed that the surface stress has quite a similar influence on the mechanical performance of the nanowires as that of the piezoelectricity. Based on the surface piezoelectricity beam model, Yan and Jiang [52] revealed that the surface piezoelectricity also plays a significant role in the bending behavior of the piezoelectric beam, which cannot be neglected. In addition, they found that the residual surface stress could soften or stiffen a piezoelectric cantilever beam depending on the stress direction and magnitude. The same authors derived the resonant frequency and the critical electric potentials for the mechanical buckling of piezoelectric nanobeams with different boundary conditions [90]. The results indicated that the mechanical behaviors of the piezoelectric nanobeams are significantly affected by the surface effects, the beam boundary conditions, the applied electrical loads and the prescribed axial strains. Li et al. [91] studied the wrinkling behavior of a piezoelectric nanofilm on a compliant substrate in the presence of the surface effects by modelling the film structure as a von Karman plate. The surface effects were found to play an important role in the wrinkling response of piezoelectric thin films as the critical electric potentials for buckling, the wavelength and the amplitudes of the wrinkles deviate significantly from the classical ones. Subsequently, based on the Kirchhoff plate theory, the surface effects on the static bending behavior of a simply supported piezoelectric nanoplate with different in-plane boundary constraints were investigated [53]. In this work, two in-plane constraints were adopted, namely the traction free conditions (Case 1) and the fixed mid-plane displacements (Case 2). For Case 1, the traction free conditions on the plate side surfaces induce a relaxation strain as $\varepsilon =-\left[e_{31}V+2\left(\sigma_{0}+e_{31}^{s}V/h\right)\right]/\left[\left(c_{11}+c_{12}\right)h+2\left(c_{11}^{s}+c_{12}^{s}\right)\right]$, with $\sigma_{0},c_{11}^{s},c_{12}^{s}$ and $e_{31}^{s}$ being the residual surface stress, surface elastic constants and surface piezoelectric constant, $c_{11},c_{12}$ and $e_{31}$ being the elastic and piezoelectric constants, *V* being the applied electric potential and *h* being the plate thickness. It is seen that the relaxation strain originally caused by the applied electric potential due to the piezoelectricity depends on the surface effects. For Case 2, the mid-plane in-plane displacements are constrained to zero, and thus induce in-plane forces, which could possibly cause the buckling of the nanoplate. This study concluded that the deflection and the electric field of the piezoelectric nanoplate in the plate thickness direction, and the in-plane relaxation strain under the mechanical and electrical loads, are size dependent due to the surface effects. By using the same modified plate model, the vibration and the buckling behaviors of piezoelectric nanoplates were investigated [92,93]. For a simply supported piezoelectric nanoplate with Case 2 in-plane constraints, it was found that the surface effects upon the vibration behavior of the plate depends on the applied electric potential, the mode number and the plate aspect ratio. It is also interesting to reveal a transition aspect ratio a/h (*a* and *h* are the length and thickness of a square piezoelectric nanoplate, respectively) by conducting the buckling analysis. At this transition point, the influence of the surface effects on the critical electrical load for buckling vanishes regardless of the value of the plate thickness *h*. The significant influence of the surface effects on the vibration [94], the critical buckling load [95] and the critical electric potential for the buckling [96] of piezoelectric nanofilms have also been observed by Zhang and his colleagues. In addition, Xu [97] presented analytical solutions for the bending deflection, the resonant frequency and the mode shape of a piezoelectric nanobeam with the consideration of shear deformation and rotary inertia. Furthermore, Zhang et al. [54] calculated the Miller–Shenoy coefficients and the natural frequencies of a piezoelectric nanoplate for pure extensional deformations based on a two-dimensional piezoelectricity theory with the consideration of the surface effects. The same authors also studied the vibration behavior of a piezoelectric shell with the nanoscale thickness [55]. Numerical results showed that the surface effects have a remarkable influence on the natural frequencies of the plates and shells with nanoscale thickness.

The flexoelectric effect upon the bending behaviors of piezoelectric cantilever, clamped–clamped and simply supported nanobeams were investigated by Yan and Jiang [72]. It was found that the beams could be either stiffened or softened depending on the boundary conditions that have been modified by the flexoelectricity. The vibration analysis of a simply supported piezoelectric nanobeam revealed that the flexoelectricity tends to reduce its resonant frequency [73]. The size-dependent bending and vibration characteristics of a clamped and a simply supported nanoplate were studied by Zhang et al. [74] and Yang et al. [98], respectively, based on the Kirchhoff plate model. In Reference [74], the flexoelectricity was found to increase the deflection while decreasing the resonant frequency of the plate. However, for the case presented in Reference [98], opposite trends of the flexoelectricity on the plate deflection and resonant frequency were observed. Recently, Liang et al. [99] studied the buckling and vibration of a piezoelectric nanofilm and found that the critical buckling load and the natural frequency of the film with the consideration of the flexoelectricity are higher than those obtained by the classical piezoelectricity plate theory.

Based on the non-local piezoelectricity theory and the conventional continuum mechanics models, Ke et al. [100,101] investigated the vibration behaviors of piezoelectric nanobeams by changing the non-local parameter, the temperature change and the external electric voltage to see their influences. The same research group further investigated the thermo-electro-mechanical free vibration of a piezoelectric nanoplate [102]. Numerical simulation results showed that an increase in the non-local parameter tends to the decrease of the vibration frequencies of the beam and the plate studied, and the non-local parameter has a significant effect on the mode shapes for the clamped–clamped and the clamped–hinged piezoelectric nanobeams.

With the consideration of both the surface effects and the flexoelectricity, the size-dependent bending properties of a piezoelectric nanobeam [85] and a piezoelectric nanoplate were studied [103]. In Reference [103], the vibration behavior of the plate was also examined. These works clearly showed that the flexoelectricity plays an indispensable role in predicting the size-dependent mechanical behaviors of piezoelectric nanostructures. Moreover, by taking into account the surface effects, the flexoelectricity and the non-local effects into a modified Kirchhoff circular plate model, the size-dependent bending and vibration behaviors of a clamped piezoelectric circular nanoplate were analyzed [104]. Simulation results indicated that the influences of the flexoelectricity and the non-local elastic effect on the plate displacements and resonant frequencies are more significant when the plate radius to thickness ratio R/h is relatively small, while such an influence of the surface effects is more prominent at larger R/h. In summary, the combined flexoelectric, surface and non-local elastic effects are significant for the whole range of the plate aspect ratio considered.

It is worth noting that there also exist a few works on modeling the flexoelectric effect upon the electromechanical coupling properties of dielectric nanostructures (without piezoelectricity). For example, the exact solutions for the displacement and the electric potential fields in a dielectric nanobeam and an elastic beam integrated with a flexoelectric nanoactuator layer were obtained by Ray [105,106], and the results revealed that the thickness of the flexoelectric nanoactuator layer can be treated as a parameter for optimizing the performance of the nanoactuator. Li et al. [107] solved the problems of static bending and free vibration of a three layer microbeam, with the middle layer being a flexoelectric dielectric layer. They adopted the piezoelectric couple stress theory [108], which considered that the size-dependent piezoelectric effect is characterized by the skew-symmetric part of rotation gradients rather than the strain gradients defined in previous works. Their results showed that, in the isotropic dielectric beam, a deformation induces polarization while an applied voltage causes deformation due to the flexoelectricity. Both the induced voltage and the deformation are size dependent. Li et al. [109] also developed a reformulated flexoelectric theory by splitting the strain gradient tensor into mutually independent parts, and the size-dependent direct and converse flexoelectric effects were captured through studying the electromechanical coupling fields of a cantilever beam, which is subjected to a concentrated force at its free end and an electric potential across its thickness. Moreover, Mao and Purohit [110] combined the theories of the strain gradient elasticity and the classical electrostatics to derive the governing equations and boundary conditions for general flexoelectric dielectrics. They solved the problems of a bending beam, a torsional shaft and a disk under pressure and predicted the corresponding size-dependent electromechanical properties and the flexoelectric modulation of the material behavior. Yan [111] further presented the exact solutions for the electromechanical responses of a dielectric nanoring subjected to both mechanical and electrical loads, with the consideration of the flexoelectricity, the surface effects and the non-local elastic effect. The results were compared with those from the theories of pure elasticity, the strain gradient elasticity and the one with the direct flexoelectricity, revealing the significant role of the surface effects and the converse flexoelectricity in the size-dependent electromechancial behavior of dielectrics.

As mentioned in the Introduction, various configurations of the nanostructures can be easily formed by using PNs with a wurtzite crystal structure. Due to the particular geometry and size, these materials may exhibit some unique properties and hold promise for applications in the biological areas and functional elements in NEMS. Therefore, it is important to investigate the size-dependent properties of these structures based on the modified continuum models. Wang et al. [112] extended the classical Kirchhoff rod model by considering the surface effects and employed the extended model to study the mechanical responses of quasi-one-dimensional nanomaterials such as nanosprings and helical nanobelts. The residual surface stresses were found to have a remarkable influence on the elastic constants of the nanosprings. Such an influence increases with the decrease of the nanospring cross-sectional radius and the increase of the nanospring helical angle. How the anisotropic surface effects influence the formation of chiral morphologies of nanomaterials was further investigated. It was demonstrated that the formation of various complicated morphologies of the nanomaterials such as the twisting and bending of nanobelts and nanohelics could be a consequence of anisotropic surface stresses [113]. Later, the same first author developed a modified Euler–Bernoulli beam model for chiral nanowires with the incorporation of both the surface effects and the material chirality [114]. The developed model was employed to investigate the bending and the buckling behaviors of the chiral nanowires. It was found that the mechanical behaviors of the nanowires were affected by the surface effects and the material chirality considerably. These works provide great insights into understanding the various structure formation and the size-dependent elastic behaviors of wurtzite PNs.

### 3.3. Size-Dependent Dynamic Performance of PNs

In order to realize the functionality of certain piezoelectric nanodevices in NEMS, some researchers also conducted investigations on the size-dependent properties of waves propagating in piezoelectric nanostructures. For example, the surface effects on the propagation of Bleustein–Gulyaev waves in a piezoelectric half-space were studied by Chen [115], and it was indicated that the Bleustein–Gulyaev wave may not exist if a fast surface layer was considered. Zhang et al. [116] investigated the anti-plane or horizontally polarized shear (SH) waves propagating in an infinite piezoelectric nanoplate. It was found that, in the presence of the surface piezoelectricity, the frequency for the anti-symmetric waves decreases while the frequency for the symmetric waves increases. Moreover, the surface effects on the wave behavior are more prominent at higher frequencies. The dispersion characteristics of elastic waves propagating in a monolayer piezoelectric nanoplate were investigated with the consideration of the surface piezoelectricity as well as the non-local effect [117]. Numerical results showed that both the non-local scale parameter and the surface piezoelectricity have remarkable influence on the size-dependent properties of dispersion behaviors. It is also found that there exists an escape frequency above which the waves may not propagate in the piezoelectric plate with the nanoscale thickness, which may have potential applications for wave band gap. The scattering of a plane harmonic compressional wave around a nanosized piezoelectric particle was studied in [118], and simulation results showed that the scattering effect of the compressional waves is significantly related to the coupling effect of the surface/interface. In addition, the surface energy was found to significantly influence the dynamic stress and electric displacement around the nano-particle. The dynamic effective properties of a piezoelectric medium with randomly distributed nano-fibers were examined to study the multiple scattering phenomena [119], which showed that the surface energy makes a significant contribution to the dynamic effective elastic modulus, especially in the region of intermediate frequencies.

## 4. Modeling of Piezoelectric Nanodevices

For the development and performance optimization of piezoelectric nanostructure-based devices, several works on the analytical modelling and analysis of piezoelectric nanoactuators and energy harvesters were carried out. Zhang et al. [120] developed anti-parallel piezoelectric bimorph nano-actuators of both cantilevered and simply supported plate types. Their numerical results showed that the deflection of the antiparallel bimorph nano-actuators was size-dependent and could achieve nearly 50 times that under the static driving voltage at the resonant frequency. In reference [54], a nano-piezoelectric plate harvester with the surface effects was presented, and the simulation results indicated that the nano-piezoelectric harvester has a stronger capability of energy conversion at the nanoscale. Based on the surface stress model, analytical solutions of an energy harvester with the flexural mode were derived, and it was demonstrated that the power density depends on the surface material constants [121]. The influence of the surface effects on the energy-harvesting performance of a piezoelectric circular nanomembrane under human blood pressure was studied and found to be more significant for a membrane with smaller thickness and larger radius-to-thickness ratio [122]. The flexoelectric effect on the performance of the nanoscale energy harvesting has also been examined in the literature. Deng et al. [123] found that the output power density and the conversion efficiency of a flexoelectric energy harvester increase significantly when the beam thickness reduces from micro to nanoscale. Recently, Wang and Wang [124] presented an analytical model for the nanoscale unimorph piezoelectric energy harvesters with the consideration of the flexoelectric effect. Their results showed that the power output could be significantly increased. For example, the maximum power output of the model with the flexoelectric effect is almost twelve times that of the classical model in some cases. Nanocomposite electrical generators based on ZnO nanowires embedded in an epoxy matrix were also modeled and quantitatively analyzed with varying aspect ratios and diameters [125,126], and the surface effects were further incorporated through a core-surface model [127]. Numerical results indicated that the maximum generated voltage is related to the diameter of nanowire and an optimum aspect ratio for each nanowire diameter was determined for the energy generator.

## 5. Conclusions

This paper provides a review on the modified continuum mechanics modeling of the size-dependent properties of PNs. In order to capture the novel properties of the piezoelectric materials at the nanoscale, the theories of the surface piezoelectricity, the flexoelectricity and the non-local piezoelectricity have been developed accordingly based on the traditional continuum mechanics frame. These modified continuum mechanics models have been widely adopted to investigate the size-dependent mechanical and physical properties of PNs, including the bending, the vibration, the buckling and the dynamic performance. A general conclusion achieved is that the surface effects, the flexoelectricity and the non-local parameter have prominent influences on these behaviors and such effects are more pronounced with the decrease of the structural size. The theoretical studies present efficient routes for quantitatively and qualitatively understanding the electromechanical and the unconventional properties of PNs. The modified continuum modeling of PN-based actuators and energy harvesters with the consideration of the size effects have also been reported, which provides a clear description of the electromechanical energy conversion and may help the design of piezoelectric nanodevices with optimal performance.

Nevertheless, some fundamental issues still remain to be resolved in future study. Firstly, the surface effects and the flexoelectricity are closely related to the curvature of the structure, thus they may have a more considerable influence on the properties of PNs with a curved surface. Therefore, the size-dependent properties of PNs with a curved surface is worth further investigation, which is not limited to the regular piezoelectric nanostructures such as piezoelectric nanocylinder and nanosphere, but a more general case. Consequently, numerical techniques with the incorporation of the size effects should also be developed. Secondly, inconsistency among the results from those modified continuum mechanics studies exists due to various small-scale effects as well as different materials chosen in the case studies. Within a certain length scale range, which small-scale effect is more dominant? There is no certain answer. Thus, a systematic and in-depth study should be conducted to give essential insights into these issues, which may need further experimental and simulation validations. Thirdly, the purpose of the theoretical studies of PNs is to serve for their potential applications. There exist quite a few prototypes of piezoelectric nanodevices, but the related theoretical works exploring the underlying mechanisms are quite limited. It is thus important to model these prototypes and clarify the size effects on their performance. Fourthly, a fully coupled electromagnetic–mechanical–thermal model with the size effects should be established to study the multi-physics coupling at the nanoscale. Lastly, dielectric elastomers have been attracting more interest in recent years due to their large deformation capability and the ease of integration into various stretchable electronic devices. When the device size is reduced to the nanoscale, the large deformation effect, together with the pseudo-piezoelectricity of the dielectrics due to the flexoelectricity, should also be investigated in future research.

## Figures and Tables

**Table 1:** Summary of the size-dependent mechanical and electromechanical coupling properties of piezoelectric nanomaterials (PNs) based on different theories. Acronyms: SPT (Surface piezoelectricity theory), TF (Theory of flexoelectricity), NPT (Non-local piezoelectricity theory), EMC (Electromechanical coupling).

Theories	Size-Dependent Properties	Materials	References
SPT	EMC fields	PZT-5H	[51,52,53,87]
SPT	bending	PZT-5H	[52,53,97]
SPT	vibration	PZT-5H	[54,55,90,92,93,94,97]
SPT	buckling	PZT-5H	[53,90,92,93,95,96]
SPT	wrinkling	PZT-5H	[91]
SPT	wave propagation	PZT-4	[115]
SPT	wave propagation	PZT-5	[116]
SPT	dynamic characteristics	PZT-4	[118]
SPT	dynamic characteristics	CoFe${}_{2}$O${}_{4}$/BaTiO${}_{3}$	[119]
TF	EMC fields	ZnO	[88]
TF	EMC fields	BaTiO${}_{3}$	[72,74,76]
TF	bending	BaTiO${}_{3}$	[72,73,74]
TF	vibration	BaTiO${}_{3}$	[73,74]
TF	bending and vibration	PZT-5H	[98]
TF	buckling and vibration	Pb(Mg${}_{1/3}$Nb${}_{2/3}$O${}_{3}$)	[99]
NPT	vibration	PZT-4	[100,101,102]
SPT and NPT	EMC fields	PZT-5H	[84]
SPT and NPT	wave propagation	PZT-5H	[117]
SPT and TF	bending	PZT-5H	[85,104]
SPT and TF	vibration	PZT-5H	[104]
SPT and TF	bending and vibration	BaTiO${}_{3}$	[103]
